# Real-world medication persistence with single versus multiple tablet regimens for HIV-1 treatment

**DOI:** 10.7448/IAS.17.4.19537

**Published:** 2014-11-02

**Authors:** Donna Sweet, Jinlin Song, Yichen Zhong, James Signorovitch

**Affiliations:** 1 Internal Medicine, The University of Kansas School of Medicine – Wichita, Wichita, KS, USA; 2 Health Economics and Outcomes Research, Analysis Group Inc., Boston, MA, USA

## Abstract

**Introduction:**

Adherence to antiretroviral (ARV) treatment for HIV-1 is crucial to achieving optimal clinical outcomes. Simplification of regimens with once-daily single-tablet regimens (STRs) can improve adherence compared to multi-tablet regimens (MTRs). This study compared real-world persistence (a proxy for treatment effectiveness and adherence) between HIV-1 infected patients receiving STRs versus MTRs.

**Materials and Methods:**

Adult HIV-1 infected patients starting their first observed ARV regimen (with at least six prior months of no ARV treatment) were identified in the MarketScan claims database (10/2008–03/2014). Persistence was measured as the time from the index regimen start date to the end of the first 90-day gap between fills for any ARV in the index regimen, or to the start date of an ARV not in the index regimen. Persistence was described using Kaplan–Meier curves and compared using log-rank tests, and Cox proportional hazards models adjusted for age, gender, insurance type, region, employment status, Charlson Comorbidity Index, other comorbidities, hospitalizations, emergency room visits and office visits. STRs were further stratified by regimen.

**Results:**

A total of 3257 patients (37%) initiated MTRs, and 5484 (63%) initiated STRs, including 4409 on efavirenz (EFV)/tenofovir (TDF)/emtricitabine (FTC), 484 on rilpivirine (RPV)/TDF/FTC, and 591 on elvitegravir (EVG)/cobicistat (COBI)/TDF/FTC. Median persistence was 45.0 months for STRs versus 15.2 months for MTRs (*P*<0.001; Figure 1). Median persistence was not reached for RPV/TDF/FTC or EVG/COBI/TDF/FTC; 31 months after RPV/TDF/FTC approval for the treatment of HIV-1 infection, more than 65% of patients who started on it remained persistent, and 19 months after EVG/COBI/TDF/FTC approval, more than 72% of patients who started on it remained persistent. Compared with MTRs, STRs had an approximately 50% lower hazard of discontinuation (adjusted hazard ratio [HR]=0.54, 95% CI 0.50–0.58). EVG/COBI/TDF/FTC and RPV/TDF/FTC had significantly longer unadjusted and adjusted persistence compared with EFV/TDF/FTC (Figure 2, Table 1).

**Conclusions:**

Among HIV-1 infected patients, the use of STRs was associated with longer regimen persistence compared with MTRs. Among STRs, EVG/COBI/TDF/FTC and RPV/TDF/FTC were associated with significantly longer persistence than EFV/TDF/FTC.

**Figure 1:**
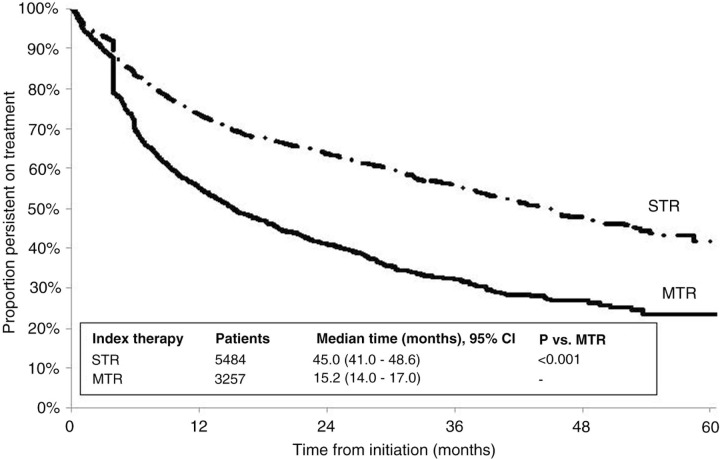
Persistence on ARV regimens among HIV-1 infected patients.

**Figure 2:**
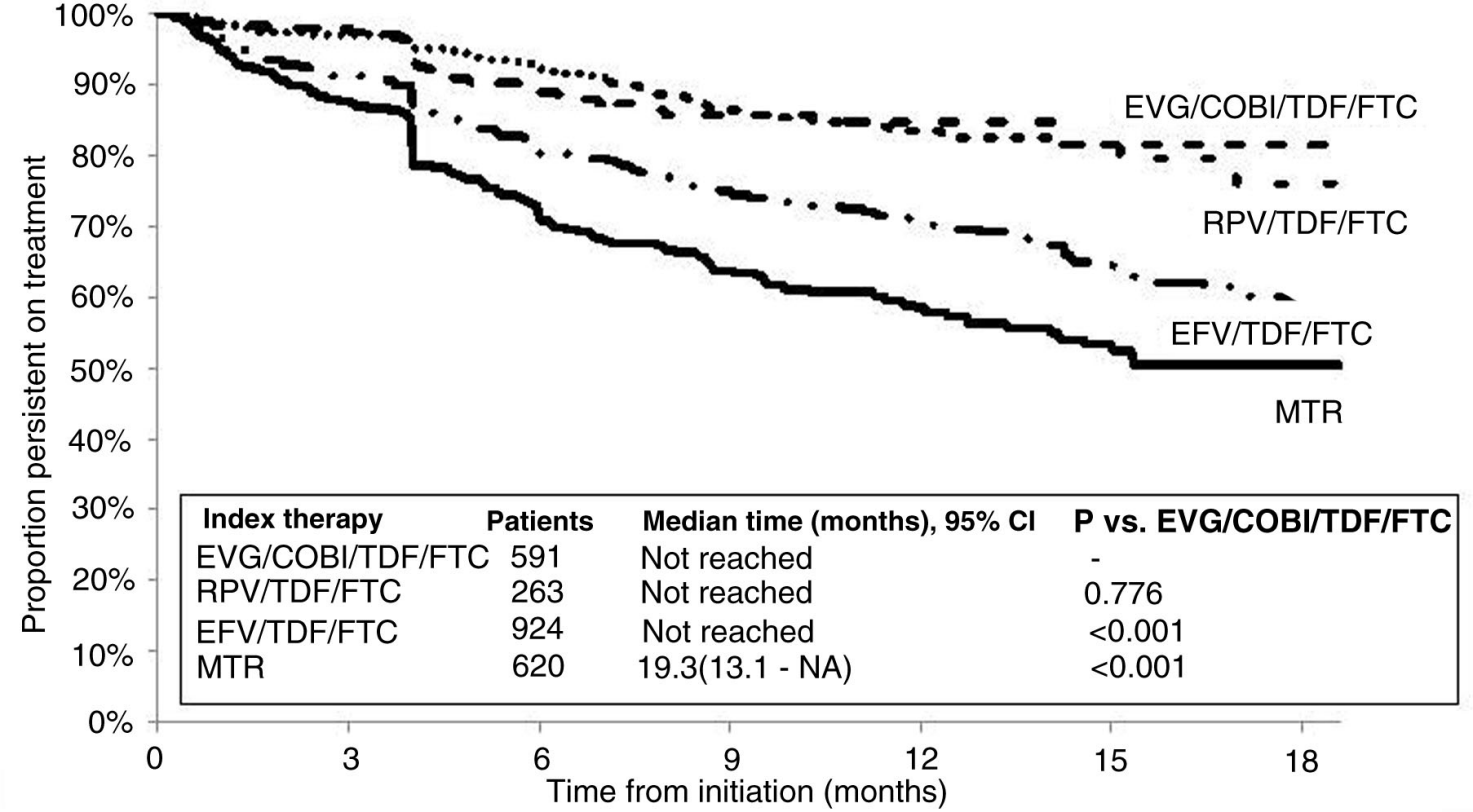
Comparison of persistence on ARV regimens among patients treated with EVG/COBI/TDF/FTC, RPV/TDF/FTC, EFV/TDF/FTC and MTR (after EVG/COBI/TDF/FTC became available on August 27, 2012).

**Table 1:** Comparison of treatment persistence between patients treated with STRs versus MTRs

Comparison	Unadjusted HR (95% CI)	Unadjusted P	Adjusted HR (95% CI)	Adjusted P
Comparison of STR vs MTR				
STR vs MTR	0.53 (0.49, 0.57)	<.0001	0.54 (0.50, 0.58)	<.0001
Pairwise comparisons of specific STRs with each other and with MTR				
EFV/TDF/FTC vs MTR	0.56 (0.52, 0.60)	<.0001	0.58 (0.53, 0.62)	<.0001
Sample restricted to patients with index date after RPV/TDF/FTC became available on August 10, 2011				
RPV/TDF/FTC vs EFV/TDF/FTC	0.67 (0.53, 0.85)	0.0009	0.66 (0.52, 0.84)	0.0007
RPV/TDF/FTC vs MTR	0.40 (0.32, 0.51)	<.0001	0.41 (0.32, 0.52)	<.0001
Sample restricted to patients with index date after EVG/COBI/TDF/FTC became available on August 27, 2012				
EVG/COBI/TDF/FTC vs EFV/TDF/FTC	0.46 (0.34, 0.61)	<.0001	0.45 (0.34, 0.60)	<.0001
EVG/COBI/TDF/FTC vs RPV/TDF/FTC	0.93 (0.60, 1.43)	0.7351	0.95 (0.61, 1.48)	0.8284
EVG/COBI/TDF/FTC vs MTR	0.30 (0.22, 0.40)	<.0001	0.31 (0.23, 0.42)	<.0001

